# Influence of Handling Conditions on the Establishment and Propagation of Head and Neck Cancer Patient Derived Xenografts

**DOI:** 10.1371/journal.pone.0100995

**Published:** 2014-06-26

**Authors:** Andrew P. Stein, Sandeep Saha, Cheng Z. Liu, Gregory K. Hartig, Paul F. Lambert, Randall J. Kimple

**Affiliations:** 1 Department of Human Oncology, University of Wisconsin Carbone Cancer Center, Madison, Wisconsin, United States of America; 2 Department of Biostatistics, University of Wisconsin Carbone Cancer Center, Madison, Wisconsin, United States of America; 3 Department of Pathology, University of Wisconsin Carbone Cancer Center, Madison, Wisconsin, United States of America; 4 Department of Otolaryngology, University of Wisconsin Carbone Cancer Center, Madison, Wisconsin, United States of America; 5 McArdle Laboratory for Cancer Research and Department of Oncology, University of Wisconsin Carbone Cancer Center, Madison, Wisconsin, United States of America; University of Pennsylvania, United States of America

## Abstract

**Background:**

Patient derived xenografts (PDXs) for head and neck cancer (HNC) and other cancers represent powerful research platforms. Most groups implant patient tissue into immunodeficient mice immediately although the significance of this time interval is anecdotal. We tested the hypothesis that the time from tumor excision to implantation is crucial for PDX passaging and establishment.

**Methods:**

We examined whether time or storage medium affected PDX viability for passaging two established HNC PDXs (UW-SCC34, UW-SCC52). Tumors were harvested, stored in ice-cold media or saline for 0–48 hours, and implanted into new mice. Tumor growth was compared by two-way ANOVA with respect to time and storage condition. Three new HNC PDXs (UW-SCC63-65) were generated by implanting patient tissue into mice immediately (Time 0) and 24 hours after receiving tissue from the operating room.

**Results:**

Similar quantities of tumor were implanted into each mouse. At the end of the experiment, no significant difference was seen in mean tumor weight between the media and saline storage conditions for UW-SCC34 or UW-SCC52 (p = 0.650 and p = 0.177, respectively). No difference in tumor formation prevalence was seen on the basis of time from harvest to implantation (≥13 of 16 tumors grew at every time point). Histological analysis showed strong similarity to the initial tumor across all groups. Tumors developed at both Time 0 and 24 hours for UW-SCC63 and UW-SCC64.

**Conclusions:**

We demonstrated that neither storage medium nor time from tumor excision to implantation (up to 48 hours) affected viability or histological differentiation in a subsequent passage for two HNC PDXs. Moreover, we revealed that fresh patient tissue is viable up to 24 hours post-resection. This information is important as it applies to the development and sharing of PDXs.

## Introduction

A well-established but now re-emerging model for human cancer is the patient derived xenograft (PDX) system. PDXs are developed by obtaining tumor samples directly from patients and subsequently implanting and passaging these tumors in immunodeficient mice [1]. The process was first documented in 1969 when Rygaard and Povlsen injected a tumor cell suspension from a patient with colon cancer subcutaneously into athymic nude mice and control mice [2]. They demonstrated greater tumor growth in the athymic mice as compared to control, leading to the use of immunodeficient mice becoming common practice for PDX development [3]. Recently, PDXs have been generated for a number of cancers including pancreatic [4]–[6], breast [7], lung [8], [9], renal [10] and head and neck [1], [11]. These studies showed that PDXs retain characteristics of the primary tumor across serial passages both at the histologic [7], [8] and molecular [1], [7], [11] levels. Daniel et al. also demonstrated that their primary small cell lung cancer PDX (only passaged in mice) retained greater similarity to the patient’s tumor as compared to a cell line generated from the same PDX [9]. Moreover, our group [1] and others [12] have successfully cryopreserved and reanimated PDXs at a later time, increasing the utility of this model system. Thus, PDXs represent a validated and reliable model for studying a variety of human cancers.

A powerful use for PDXs is the testing of standard and novel treatments in an *in vivo* system with greater heterogeneity than genetically engineered mouse models and a more relevant tumor microenvironment than cell lines [1], [4], [11]. Once established, PDXs are amplified *in vivo*, injected into numerous mice, and the mice are subsequently stratified into different treatment groups. The capacity of a specific treatment regimen to slow or halt tumor growth can be assessed by comparing the mean tumor growth over time for each group as compared to control (untreated) mice. In this manner, investigators can evaluate the effect of many alternate therapies on one specific tumor type derived from a single patient. Additionally, molecular alterations induced by the different treatments can be analyzed by harvesting post-treatment tumor samples either by flash freezing tumor chunks in liquid nitrogen for genome wide studies or fixing tumors in formalin followed by paraffin embedding for biomarker analysis. It has also been argued that this model system could be employed in personalized cancer therapy by generating PDXs from a patient’s cancer, testing a variety of chemotherapeutics on mice bearing their tumor and then selecting a new treatment regimen for the patient based on these *in vivo* results [13].

There are a number of variations in the procedures used to establish PDXs. For example, some groups describe surgical implantation of small (2–3 mm) tumor chunks into the flanks [14] while others mince the tumors to create a cell suspension and inject the suspension subcutaneously through a large gauge needle [15]. Furthermore, certain groups only use athymic nude mice [3], others use non-obese diabetic severe combined immunodeficiency (NOD-SCID) mice [7] and some employ both strains [1]. Significantly, these different techniques have all led to successful tumor growth. Another recurring theme that emerges in PDX development is that tumors are taken as quickly as possible (at most within 3 hours) from the time of biopsy and then injected into the immunodeficient mice [1], [2], [8], [10], [16]. Our group and others proceed in this manner due to the belief that the time from tumor excision to xenograft implantation is important. However, there is no information in the literature to suggest that tumor chunks must be injected/implanted as soon as possible into the mice [17]. In addition, while mouse-to-mouse passage is typically performed as rapidly as possible, there is again, little data to support the necessity of this practice.

Due to the uncertainty regarding optimal harvesting and implantation techniques, we undertook this study to determine whether there was a relationship in the growth potential of the PDX based on the time delay from initial tumor excision to its ultimate implantation in the mice. In addition, we sought to determine whether the storage medium used during the processing delay affected PDX viability. This work is important since there are a number of factors outside of the researcher’s control that can influence the ability to obtain tumor biopsies from consented patients in a timely manner. Moreover, once PDXs are established, re-implantation requires the availability of recipient mice and their dissemination to other laboratories may require shipment. Both of these issues can lead to delays in re-implantation. Our results demonstrate the tumor is still viable and capable of growing in the next generation of mice up to at least 48 hours after the initial tumor excision and that the nature of the storage solution, be it tissue culture medium or saline solution, has no effect on tumor growth. Furthermore, we revealed that fresh patient tissue from the operating room (OR) is viable and can be used to establish a new PDX up to at least 24 hours after initial excision from the patient. The ability to delay tumor implantation has important implications with regard to sharing of these resources as well as the logistics of initial PDX establishment and subsequent maintenance.

## Materials and Methods

### Mice

Six to eight week old male and female NOD-SCID gamma (NSG, NOD.Cg-*Prkdc^scid^ Il2rg^tm1Wjl^*/SzJ) mice (purchased from Jackson Laboratories) were used for PDX development and amplification. All mice were kept in the Association for Assessment and Accreditation of Laboratory Animal Care-approved Wisconsin Institute for Medical Research (WIMR) Animal Care Facility. Animals were housed in specific pathogen free rooms, and they lived in autoclaved, aseptic, and microisolator cages with a maximum of four animals per cage. Food and water were provided ad libitum. All studies involving the mice were carried out in accordance with an animal protocol approved by the University of Wisconsin (Protocol Number: M02518). During the experiments, each mouse’s weight and clinical health were evaluated on a weekly basis.

### Previously Established Patient Derived Xenografts

Our PDXs were derived from patients with newly diagnosed or recurrent head and neck cancer (HNC) who completed a written consent in accordance with an IRB approval from the University of Wisconsin. We previously described the establishment of our PDX model in greater detail [1]. As tumor samples from the OR often provide only enough tissue for implantation into two to four mice, the main studies described in this manuscript utilized established PDXs at an early passage. Passaging and implantation of PDXs was accomplished by first harvesting tumors from NSG mice bearing the tumor of interest, transferring the tumor in a sterile fashion to a 2.0 mL eppendorf tube with a 1∶1 mixture of media (Dulbecco’s Modified Eagle Medium with 10% fetal bovine serum, 1% penicillin/streptomycin, and 2.5 µg/mL amphotericin B) and matrigel (catalog #354230, BD Biosciences, Inc) and mincing the tumor into less than 1 mm^3^ pieces. Finally, the tumor suspension was drawn into a 1 mL syringe through an 18-gauge needle and injected subcutaneously into two flanks of NSG mice.

### Experimental Design

**Figure 1:**
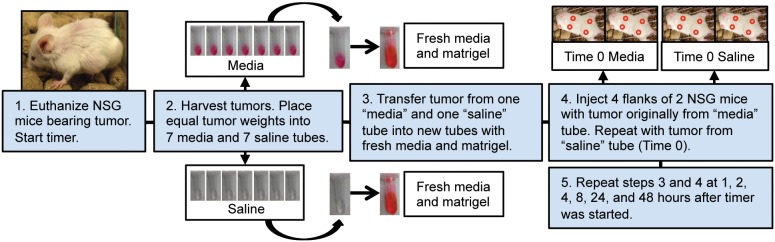
Experimental Methods. Flow diagram depicting the experimental setup.

Two PDXs that demonstrated sustained growth in early passages (UW-SCC34 and UW-SCC52) were selected for this experiment. The process was carried out separately for the UW-SCC34 and UW-SCC52 groups, but an identical protocol was followed both times. First, four NSG mice bearing two tumors each of the PDX of interest were euthanized, and a timer was started when death was confirmed. All tumors were harvested from the four mice at the same time. Pooled tumor chunks were weighed and divided equally across fourteen 2.0 mL eppendorf tubes pre-conditioned on ice: seven containing media and seven with saline. Additionally, one tumor chunk (referred to as the pre-implantation sample) was fixed in 10% neutral buffered formalin for 48 hours and paraffin embedded for later histological analysis. Next, one media+tumor tube and one saline+tumor tube were removed from the ice. Each tumor chunk was transferred to its own, new tube with a 1∶1 mixture of fresh media and matrigel, minced into 1 mm^3^ pieces, and injected into four flanks of two NSG mice (n = 8). The time was recorded after all four mice were injected (Time 0, 40 minutes). In this manner, we ended up with two groups at this time point: “Time 0 Media” and “Time 0 Saline”. Both groups were comprised of two NSG mice each with four injection sites. Thus, each group had the potential to develop eight tumors (Figure 1). This process was repeated at 1, 2, 4, 8, 24, and 48 hours after the timer was started. In the interim, the tumors in media or saline were maintained at 4**°**C.

Tumors were allowed to grow for 3 weeks for UW-SCC34 and 6 weeks for UW-SCC52. The duration of tumor growth was dictated by tumor growth kinetics and the health of the mice. At the end of each experiment, tumors from every mouse were harvested, weighed, and photographed (harvested tumors 1 and 2 days later for the 24 hour and 48 hour groups, respectively). Each tumor was fixed in 10% neutral buffered formalin for 48 hours and then paraffin embedded. One tumor was randomly selected from each of the fourteen groups for further histological analysis.

### Histology

Tumor blocks were sectioned (5 µm) and hematoxylin and eosin (H&E) stains were carried out on every fifth section. All slides were imaged on an Olympus BX51 microscope (Olympus America, Inc). Comparisons were made between the histology of the pre-implantation sample and the tumors grown in the next generation of NSG mice. The similarity in histology of tumors across the fourteen different groups within the two experiments was also assessed. A board certified pathologist specializing in diseases of the head and neck (C.Z.L.) carried out the histological analysis to define differentiation, keratinization, necrosis/cystic change and infiltrative pattern for each tumor.

### New Patient Derived Xenografts

Once we determined that neither the time from excision to re-implantation nor storage medium affected PDX growth in a subsequent passage, we performed a variation of this experiment utilizing fresh patient tissue from the OR. Three new patients with HNC were consented to donate tissue for PDX establishment (UW-SCC63, UW-SCC64, UW-SCC65). Due to the small amount of tumor we receive from the OR, there could only be two groups for these experiments. Therefore, we focused on the time factor for initial PDX establishment. When we received the fresh patient tissue, it was weighed and distributed evenly into two tubes with media. For one tube, matrigel was added immediately and the tumor was minced and injected into four flanks of two NSG mice (n = 8, Time 0). The other tube (with tumor and media) was stored at 4**°**C for 24 hours. Then, the tumor was transferred to a new tube with fresh media and matrigel, and the tumor was minced and injected into four sites of two NSG mice (n = 8, 24 hours). This same process was repeated for all three new patient samples.

After nine weeks, tumor growth was assessed and compared between the Time 0 and 24 hour groups for each new PDX. Only UW-SCC64 had tumors at an appropriate size for passaging. Therefore, all mice in the UW-SCC64 experiment were euthanized, and the tumors were excised, weighed and photographed (harvested tumors 1 day later for the 24 hour group). For UW-SCC63 and UW-SCC65, mice were palpated for tumor growth at each injection site.

### Statistical Analysis

We powered this study to detect a difference in PDX viability between the first and last time points (Time 0 and 48 hours, respectively). There does not exist any information in the literature about the effect of time on PDX viability and growth, so we did not have any information to guide our estimation for the difference we expected to see between these groups. Therefore, we conservatively estimated there would be a 50% decrease in tumor formation at 48 hours as compared to Time 0. To detect a 50% decrease in tumor formation at a significance level of 0.05 and a power of 0.80, we calculated a sample size of 8 per group [18]. In order to evaluate the effect of storage medium as well, we required a sample size of 8 at each time point for both the media and saline groups.

For the UW-SCC34 and UW-SCC52 experiments, all tumors were individually weighed at the end. The tumor weights obtained for these PDXs from the two growth mediums (media and saline) were summarized using descriptive statistics including mean and standard deviation. Tumor weights across the two growth mediums were compared using the Snedecor F-test from a two-way analysis of variance (ANOVA) model that included time as a factor. Hence this model adjusted for the time when the sample was implanted into the mouse. The difference in the mean tumor weights between the media and saline growth medium samples was calculated with a 95% confidence interval. These statistical analyses were performed using SAS version 9.2.

For the newly established PDX experiments, all tumors were harvested and weighed from the UW-SCC64 PDX. Mean tumor weights for the Time 0 and 24 hour groups were compared using a two-sample t-test with equal standard deviations. This analysis was carried out using Graphpad Prism v6.0d. For all statistical analyses a p-value less than 0.05 was considered statistically significant.

## Results

### Mice

At baseline, the mice weighed an average of 22.8 grams (g) (range 19.0 g–26.8 g). They were free of any microorganisms and had not been subjected to any prior treatment or testing.

### Pre-implantation Tumor Weights

**Figure 2:**
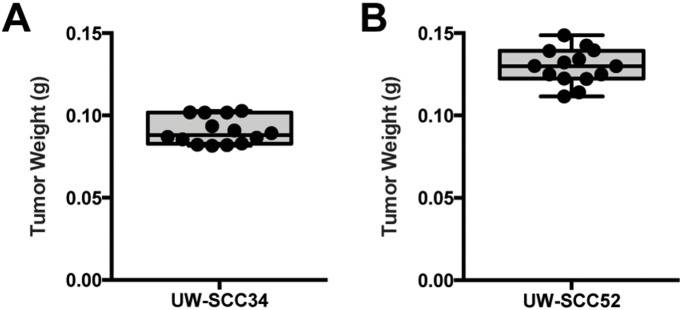
Pre-implantation Tumor Weights. (A) Boxplot for the fourteen UW-SCC34 pre-implantation tumor weights demonstrates the absence of any outliers. (B) Boxplot of the pre-implantation tumor weights for UW-SCC52 also shows that there are not any outliers.

To minimize differences in the amount of tumor implanted in every group, we first distributed tumors such that equal weights were injected into the mice at each time point and for each storage condition (Figure 1). For the fourteen UW-SCC34 groups, the mean pre-implantation tumor weighed 0.0906 g (range 0.0816 g–0.1027 g), and for UW-SCC52 the mean was 0.1298 g (range 0.1116 g–0.1487 g). As depicted by the boxplots in Figures 2A and 2B, there were no outliers among the pre-implantation tumor weights for the fourteen groups in either experiment.

### Effect of Storage Medium and Time on PDX Maintenance

**Figure 3:**
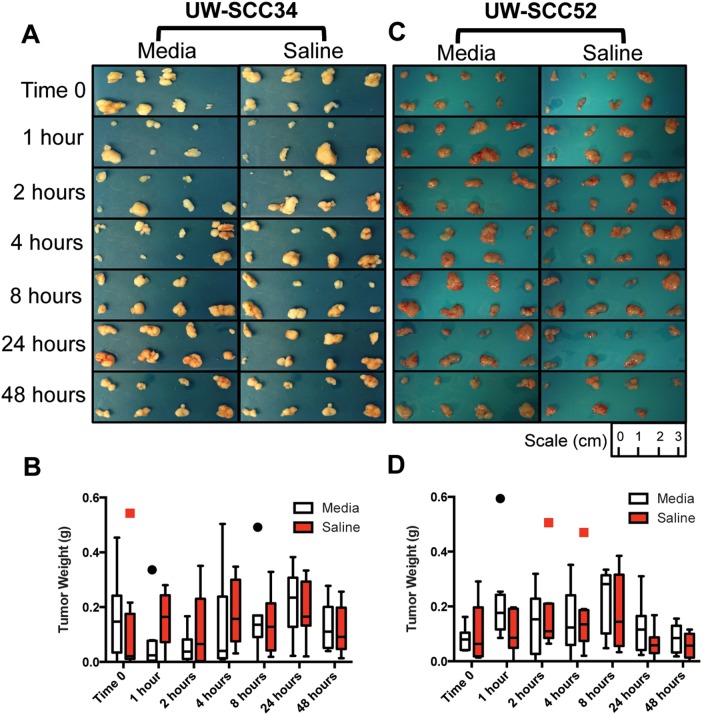
End of Experiment Tumor Pictures and Weights. (A) Photographs of all tumors harvested at the end of the experiment for UW-SCC34. (B) Boxplots for the end of experiment tumor weights for the UW-SCC34 experiment distinguished by storage medium and time. (C) Photographs of all tumors harvested at the end of the experiment for UW-SCC52. (D) End of experiment tumor weights for UW-SCC52 also separated by storage medium and time.

At the end of both experiments, all tumors were harvested, photographed (Figures 3A and 3C) and weighed from the 28 mice. First, we examined whether the storage medium (media versus saline) affected tumor growth potential. For UW-SCC34 the mean tumor weight for all tumors in the media group (n = 56) was 0.132 g (standard deviation (SD) 0.130 g), and for the saline group (n = 56) the mean was 0.142 g (SD 0.120 g). The difference between the mean tumor weights for these two groups was −0.010 g (95% CI: −0.056 g, 0.035 g), which was not statistically significant. In order to account for any relation to the time when the tumor was implanted, we carried out a two-way ANOVA with storage medium and time as the factors. This also demonstrated that there was no statistically significant difference between the mean tumor weight in the media and saline groups (p = 0.650, Figure 3B). We carried out the same analyses for the UW-SCC52 experiment. For all tumors in the media group (n = 56) the mean weight was 0.146 g (SD 0.113 g) and for the saline group (n = 56) the mean was 0.119 g (SD 0.111 g). Once again, with a difference between the means of 0.027 g (95% CI: −0.012 g, 0.067 g), there was no significant difference between the mean tumor weights based on storage medium. Finally, utilizing a two-way ANOVA, there was no significant difference between tumor weights for the media versus saline storage mediums when taking time into account (p = 0.177, Figure 3D). Thus, based on our replicate experiments the storage medium did not have any effect on tumor growth potential even when accounting for time from tumor excision to re-implantation.

We also assessed if the time from tumor excision to implantation had any effect on tumor viability. At each time point there were 16 potential tumors that could have developed (8 from media and 8 from saline group). Based on Table 1, for the UW-SCC34 experiment it is evident that time did not impact the ability for the PDX to grow, because at least 13 of 16 tumors developed at each of the seven time points. Interestingly, at the later time points, 24 and 48 hours, 16 of 16 PDXs grew. Similarly, for the UW-SCC52 experiment, at least 15 of 16 tumors developed at each time (Table 1). Thus, the length of delay before re-implantation did not impact tumor viability for either the UW-SCC34 or UW-SCC52 experiments, within the time frame studied (i.e. up to 48 hours of storage at 4**°**C). Moreover, there was no relationship between the pre-implantation tumor weight and ultimate tumor development (ie: the few mice that failed to develop one or two tumors did not have the lowest pre-implantation tumor weights). This further indicated that the amount of tumor injected at the start of the experiments was appropriate to promote subsequent tumor growth.

**Table 1:** Number of tumors that developed at each time point.

	UW-SCC34		UW-SCC52	
	Media	Saline	Media	Saline
**Time 0**	7/8	7/8	8/8	8/8
**1 hour**	6/8	7/8	8/8	7/8
**2 hours**	7/8	7/8	7/8	8/8
**4 hours**	8/8	8/8	7/8	8/8
**8 hours**	7/8	8/8	8/8	8/8
**24 hours**	8/8	8/8	8/8	7/8
**48 hours**	8/8	8/8	8/8	7/8

### Histology

**Figure 4:**
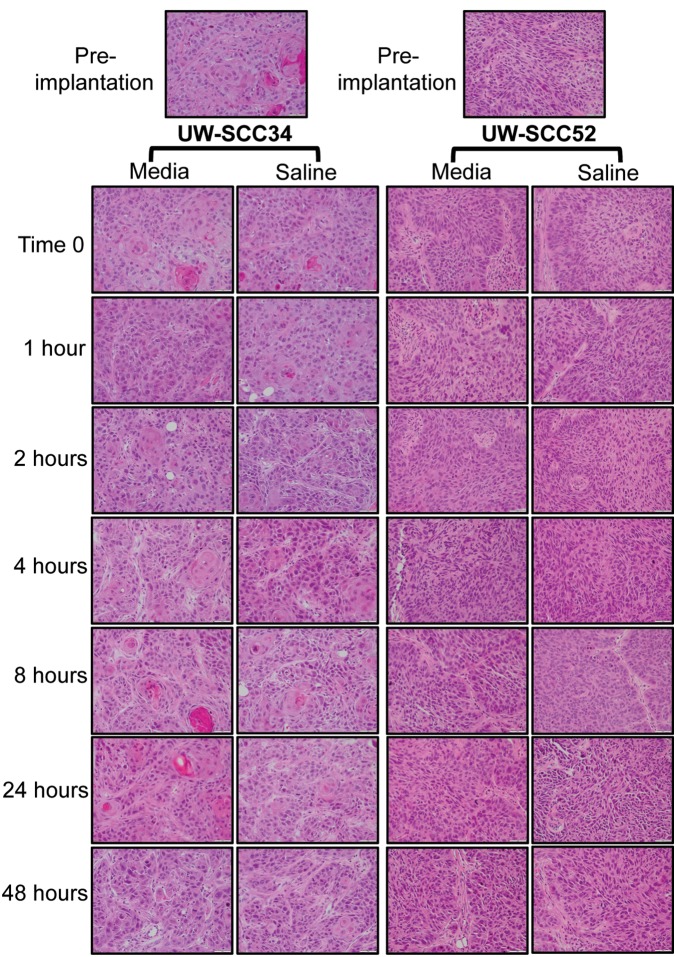
H&E Slides for Pre-implantation and End of Experiment Tumors. Representative images from the H&E stained slides for each group within the UW-SCC34 and UW-SCC52 experiments.

As displayed in Table 2, the pre-implantation UW-SCC34 tumor was moderately differentiated with 10% keratinization, 5% necrosis/cystic change and an infiltrative pattern on H&E staining. Importantly, all tumors in the subsequent passage from the seven different time points and two storage mediums demonstrated the same characteristics of moderate differentiation with an infiltrative pattern along with some keratinization (range <5%–15%) and necrosis/cystic change (range <5%–15%). Representative images from the stained slides are shown in Figure 4. Next, the UW-SCC52 pre-implantation tumor was evaluated and had moderate differentiation, no keratinization, 20% necrosis/cystic change and an infiltrative pattern (Table 3). Once again, the histology of the tumors from the next passage (all time points and both storage mediums) was quite similar with each displaying moderate differentiation, no keratinization, some necrosis/cystic change (range 5%–25%) and an infiltrative phenotype. Thus, histologically we have demonstrated that the same tumor developed regardless of the time or storage medium for both UW-SCC34 and UW-SCC52.

**Table 2:** Histological classification of UW-SCC34 tumors.

	Differentiation	Keratinization	Necrosis/Cystic Change	Infiltration
**Pre-implantation**	Moderate	10%	5%	Yes
**Time 0 Media**	Moderate	10%	5%	Yes
**Time 0 Saline**	Moderate	5%	15%	Yes
**1 hour Media**	Moderate	5–10%	5%	Yes
**1 hour Saline**	Moderate	<5%	5%	Yes
**2 hours Media**	Moderate	5%	10–15%	Yes
**2 hours Saline**	Moderate	10%	<5%	Yes
**4 hours Media**	Moderate	5%	10%	Yes
**4 hours Saline**	Moderate	10%	10%	Yes
**8 hours Media**	Moderate	10%	<5%	Yes
**8 hours Saline**	Moderate	5%	5%	Yes
**24 hours Media**	Moderate	15%	5%	Yes
**24 hours Saline**	Moderate	5%	5%	Yes
**48 hours Media**	Moderate	<5%	<5%	Yes
**48 hours Saline**	Moderate	5%	<5%	Yes

**Table 3:** Histological characterization of UW-SCC52 tumors.

	Differentiation	Keratinization	Necrosis/Cystic Change	Infiltration
**Pre-implantation**	Moderate	No	20%	Yes
**Time 0 Media**	Moderate	No	20%	Yes
**Time 0 Saline**	Moderate	No	10%	Yes
**1 hour Media**	Moderate	No	15%	Yes
**1 hour Saline**	Moderate	No	10%	Yes
**2 hours Media**	Moderate	No	5%	Yes
**2 hours Saline**	Moderate	No	25%	Yes
**4 hours Media**	Moderate	No	10%	Yes
**4 hours Saline**	Moderate	No	25%	Yes
**8 hours Media**	Moderate	No	20%	Yes
**8 hours Saline**	Moderate	No	5%	Yes
**24 hours Media**	Moderate	No	20%	Yes
**24 hours Saline**	Moderate	No	5%	Yes
**48 hours Media**	Moderate	No	5%	Yes
**48 hours Saline**	Moderate	No	5%	Yes

### Effect of Time on new PDX Establishment

We investigated whether a 24 hour delay in the implantation time (tumor stored in media at 4**°**C during the delay) had any effect on PDX establishment for three new HNC PDXs (UW-SCC63, UW-SCC64, UW-SCC65). Approximately equal tumor volumes were injected into the two mice at Time 0 and 24 hours for each of the three PDXs. For UW-SCC63 the pre-implantation tumor weights were 0.0694 g and 0.0723 g while the pre-implantation weights were much lower for UW-SCC64 (0.0115 g and 0.0117 g) and UW-SCC65 (0.0085 g and 0.0093 g).

**Figure 5:**
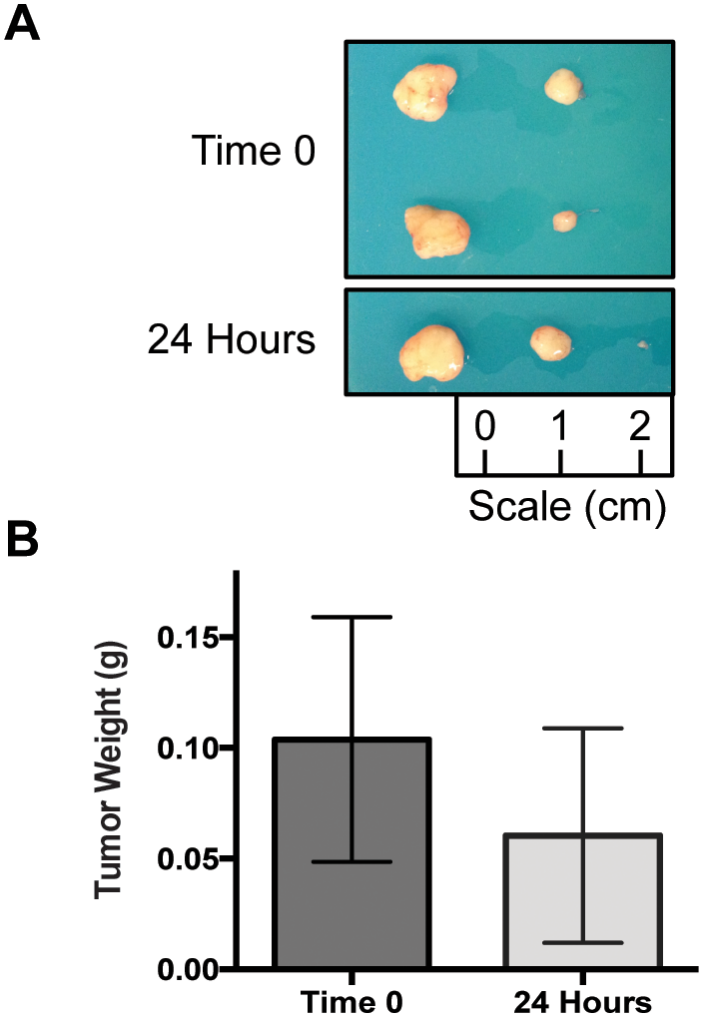
Tumor Pictures and Weights for UW-SCC64. (A) Photographs of all tumors harvested at the end of the experiment for UW-SCC64. (B) Bar graphs depicting the mean tumor weights for the Time 0 and 24 hour groups in the UW-SCC64 experiment.

Nine weeks after initial implantation, the mice bearing each new PDX were evaluated for tumor growth. Only mice with the UW-SCC64 PDX had sufficient tumor volume for passaging. Therefore, these mice were euthanized, and all tumors were individually photographed and weighed (Figures 5A and 5B). The mean weight for the Time 0 tumors was 0.104 g (SD 0.156 g) and at 24 hours the average was 0.060 g (SD 0.137 g). There was no statistically significant difference between the mean tumor weights between these two groups (p = 0.564). Moreover, there was a similar number of tumors that arose in each of the two groups. Four of eight tumors grew at Time 0 and three of eight developed in the 24 hour group. For UW-SCC63, there were three palpable tumors in the Time 0 group and two in the 24 hour cohort. On the other hand, UW-SCC65 did not demonstrate any palpable tumors in either group. Overall, it appears in the PDXs that were able to establish themselves, the 24 hour time delay did not affect PDX viability. Interestingly, the pre-implantation tumor weight did not appear to have an important effect on subsequent tumor growth since the UW-SCC64 tumors (which demonstrated the greatest growth) had approximately six times less tumor implanted initially as compared to UW-SCC63.

## Discussion

PDXs represent an important and validated model for the investigation of numerous different cancer subtypes, especially in the trial of novel and standard chemotherapeutics. Because patient tissue is precious and often difficult to obtain, investigators must take great care to optimize this model system. For this reason we wanted to further explore the potential boundaries of PDX passaging by testing the hypotheses that 1) time from tumor excision to ultimate implantation in new NSG mice is important and 2) storage medium has an effect on PDX viability and growth. Surprisingly, we found that neither the storage medium (media or saline) nor time of storage (up to 48 hours at 4**°**C) affected PDX growth and establishment in a subsequent passage. Furthermore, we revealed that delaying the implantation of fresh patient tissue up to 24 hours (tumor stored in ice-cold media during delay) did not affect initial PDX establishment.

To fully appreciate the importance and necessity of PDXs in the field of cancer research, it is valuable to assess other available models along with their strengths and pitfalls. First, cell lines have played an important role in the history of oncologic research and drug discovery. HeLa cells represent the first human cancer cells grown in the laboratory, and analyses of these cells and other cell lines that followed have provided much of our current molecular understanding of cancer [19]. Furthermore, the standard pre-clinical process for investigating novel cancer chemotherapeutics by the National Cancer Institute (NCI) begins with assessment of *in vitro* activity in 60 established cancer cell lines (NCI-60) followed by *in vivo* assessment of these cell lines in mice through both the hollow fibre assay and xenografts [20], [21]. Recently, the validity of utilizing cell lines as surrogates for the primary tumor has been brought into question [22]. Gillet et al. demonstrated through genetic analyses that no correlation existed between patient tumors and established cancer cell lines, and in fact the cell lines bore greater resemblance to each other than to clinical samples [23]. Additional groups have also revealed key differences between primary tumors and derived cell lines [24]. However, investigators also note that important pathway changes still exist in these tumor cells [25]. Thus, despite differences with respect to the primary tumor, genetic expression profiles can be employed to select cell lines for specific analyses [26]–[28].

Due to the growing concern over the representativeness of cell lines to the primary cancer, there is the need for additional models to supplement this work. Transgenic mouse models represent another distinct system that have been utilized in cancer research. These models are most often used to assess the impact of relevant oncogenic mutations to tumorigenesis and therapeutic response for a variety of human cancers including pancreatic ductal adenocarcinoma [29], [30], soft-tissue sarcoma [31], lung adenocarcinoma [32], HNC [33] and many others. The scope of questions that can be examined by transgenic mice is quite broad as exemplified by prior studies on transgenic mice expressing human papillomavirus (HPV) oncogenes in which interactions between these specific oncogenes with fanconi anemia deficiency genes [34], estrogen and cervical cancer progression [35], and oncoprotein expression in relation to lymphocyte trafficking [36] have been elucidated. All in all, transgenic mouse research can provide unique insight into mechanisms of carcinogenic mutations and potential therapeutic interventions. However, these models tend to have powerful driving oncogene mutations and other very specific genetic changes that may limit the scope of their applicability to clinical oncology.

For the PDX model, each patient’s cancer is unique and represents countless genetic changes that accrued over the time the cancer developed. These cells are not intentionally transformed or forced to overexpress specific proteins like cell lines and transgenic mouse models, respectively. Ideally this means PDXs should more closely resemble the primary tumor. As described in the introduction, many groups have validated the utility of this system, including the capacity for PDXs to recapitulate the metastatic potential of the primary tumor [7], [37]. Importantly, Tentler et al. recently reviewed the translational track record for PDXs in oncology drug development for a wide range of cancers, and the future for PDXs in the development of predictive biomarkers for clinical trials appears promising [17].

PDXs can be injected into mice in either an orthotopic or heterotopic manner [17]. The PDXs that we have generated, utilized and described throughout this paper were implanted in a heterotopic fashion since HNCs were grown subcutaneously in the mice. Other groups studying pancreatic [4], [6], lung [9], [10], and renal [10] cancers have also utilized subcutaneous tumor implantation for their work. Subcutaneous tumors allow for easier access for size measurements by calipers during therapeutic studies. Moreover, tumors can be grown to a much larger volume subcutaneously as compared to orthotopic sites before causing harm to the mice. In this manner, subcutaneous tumors allow for greater amplification of this valuable tissue, which can then be harvested for histological as well as molecular analyses and for passaging to new mice. Orthotopic implantation, whereby tumor cells are injected at the site of origin, represent another viable manner to propagate and study PDXs. This technique has been described for breast [7], brain [38], and HNCs [37], [39]. It is thought that this model allows tumors to develop in a more representative microenvironment [39]. Indeed this system is especially useful for studying metastasis [7], [37]. However, in particular for the head and neck region, only small tumor volumes can be generated before causing harm to the mice. Qiu et al. documented that after two weeks mice with oral PDXs required modified diets due to difficulty eating [37]. Owing to these small tumor sizes, it is difficult to propagate HNC PDXs grown orthotopically. It is clear that heterotopic and orthotopic PDXs both have unique advantages and disadvantages for modeling human cancer.

We believe that each of the oncologic models discussed as well as others that are available can and should play a role in future research [17]. Indeed it is important to define the spectrum of utility for every system and determine how each can be maximized to answer specific questions. For example, novel, targeted therapeutics could be initially employed in transgenic mice that are made to overexpress the targeted protein. This information could then translate into screening PDXs for the protein of interest, assessing their relative response to the novel drug, and identifying predictive biomarkers. Even within a specific model system, such as the PDXs, it is important to employ the spectrum of available methods including both heterotopic and orthotopic tumors. Orthotopic tumors appear superior for studying metastasis, especially in the head and neck region, while subcutaneous tumors allow for easier evaluation of therapeutic response with less harm to the mice. In this manner, we feel the strengths of each model can be exploited to advance the treatment of human cancers.

Although our methods were carefully planned and executed, there are, of course, limitations to this work. First, we did not carry out the full experimental protocol on fresh patient tissue owing to the small quantity of tumor we receive from the OR. Therefore, we would not have been able to make the number of injections necessary to carry out this work. Instead we performed a smaller experiment with three new PDXs to determine if they retained viability up to 24 hours after initial excision from the patient. Next, we did not perform this experiment on each of our PDXs, but we did select two that represent the broad spectrum of growth that we have seen among our cohort of PDXs. While the histological characterization we performed demonstrated no differences based on time or storage medium (Tables 2 and 3, Figure 4), molecular changes such as gene methylation or the development of mutations could occur as the time increases between tumor excision and implantation. However, we did not carry out specific analyses to address this potential issue. Finally, we cannot say how much time beyond 48 hours the tumors would remain viable for passaging.

Despite the limitations of this experiment, our results are surprising as most researchers in the PDX community, including us, believed strongly in the importance of the time interval to implantation. Therefore, we did not expect the fresh patient tissue to remain viable 24 hours post-resection nor did we anticipate the passaged PDXs (UW-SCC34 and UW-SCC52) would retain equivalent viability across the seven different time points, especially up to 48 hours. These experiments represent the first non-anecdotal evidence in the literature about the relationship between time, storage medium and ultimate PDX development. We hope other investigators can apply our findings to their PDX work in order to maximize the potential of this valuable model system. For example, it is difficult to perfectly monitor the health of immunodeficient mice since they are very fragile and within a short window they can change from clinically healthy to on the verge of death. Thus, planning ahead for passaging PDXs is not always trivial, but with our data, researchers can now feel comfortable harvesting tumor, storing it in either ice-cold media or saline up to 48 hours, and then passaging the tumor when new mice arrive. This process has already been implemented successfully in our lab. Similarly, if any miscommunication arises in obtaining fresh patient tissue from the OR, we believe it is worthwhile to implant the tissue at whatever time it is eventually received since this primary tissue is so limited, and it is a reasonable to suggest the primary tumor would retain its viability past 24 hours. Lastly, our work will increase the ability of laboratories to share PDXs since now researchers at one institution can harvest fresh tumor and ship it overnight to another lab without needing to go through the process of slow freezing the tumor in dimethyl sulfoxide or glycerol first [1]. Overall, we hope that our work will open the doors a bit wider for PDX establishment and propagation in order to increase the overall benefit of this model system.

## Supporting Information

Checklist S1 This checklist provides a summary of the locations in the manuscript where we have reported specific data based on the recommendations from the Animal Research: Reporting In Vivo Experiments (ARRIVE) guidelines.
(PDF)
